# Genetic differentiation and inferred dynamics of a hybrid zone between Northern Spotted Owls (*Strix occidentalis caurina*) and California Spotted Owls (*S. o. occidentalis*) in northern California

**DOI:** 10.1002/ece3.3260

**Published:** 2017-07-27

**Authors:** Mark P. Miller, Thomas D. Mullins, Eric D. Forsman, Susan M. Haig

**Affiliations:** ^1^ U.S. Geological Survey Forest and Rangeland Ecosystem Science Center Corvallis OR USA; ^2^ USDA Forest Service Pacific Northwest Research Station Corvallis OR USA

**Keywords:** California Spotted Owl, genetic structure, hybrid zone, microsatellites, mtDNA, Northern Spotted Owl, *Strix occidentalis*

## Abstract

Genetic differentiation among Spotted Owl (*Strix occidentalis*) subspecies has been established in prior studies. These investigations also provided evidence for introgression and hybridization among taxa but were limited by a lack of samples from geographic regions where subspecies came into close contact. We analyzed new sets of samples from Northern Spotted Owls (NSO:* S. o. caurina*) and California Spotted Owls (CSO:* S. o. occidentalis*) in northern California using mitochondrial DNA sequences (mtDNA) and 10 nuclear microsatellite loci to obtain a clearer depiction of genetic differentiation and hybridization in the region. Our analyses revealed that a NSO population close to the northern edge of the CSO range in northern California (the NSO Contact Zone population) is highly differentiated relative to other NSO populations throughout the remainder of their range. Phylogenetic analyses identified a unique lineage of mtDNA in the NSO Contact Zone, and Bayesian clustering analyses of the microsatellite data identified the Contact Zone as a third distinct population that is differentiated from CSO and NSO found in the remainder of the subspecies' range. Hybridization between NSO and CSO was readily detected in the NSO Contact Zone, with over 50% of individuals showing evidence of hybrid ancestry. Hybridization was also identified among 14% of CSO samples, which were dispersed across the subspecies' range in the Sierra Nevada Mountains. The asymmetry of hybridization suggested that the hybrid zone may be dynamic and moving. Although evidence of hybridization existed, we identified no F1 generation hybrid individuals. We instead found evidence for F2 or backcrossed individuals among our samples. The absence of F1 hybrids may indicate that (1) our 10 microsatellites were unable to distinguish hybrid types, (2) primary interactions between subspecies are occurring elsewhere on the landscape, or (3) dispersal between the subspecies' ranges is reduced relative to historical levels, potentially as a consequence of recent regional fires.

## 1 INTRODUCTION

Studies of hybrids can provide new insights regarding interactions of species. Hybridization may occur when the distributions of two taxa come into secondary contact (Barton & Hewitt, 1985), although the specific outcomes of hybridization can vary (Abbott et al., 2013; Arnold, 1997; Moore, 1977; Todesco et al., 2016). Birds in particular are known to have high interspecific hybridization rates (Grant & Grant, 1992), with up to 16% of species demonstrating evidence of hybridizing in the wild (Ottenburghs, Ydenberg, Van Hooft, Van Wiren, & Prins, 2015). These estimates, however, reflect rates on a per‐species basis and do not reflect the overall frequency of hybridization in populations where it occurs. Thus, detailed investigations of hybrid zones and hybridization provide unique opportunities to better quantify the magnitude of direct interactions between taxa and further elucidate the specific dynamics of the hybridization process.

In this study, we focus on analyses of two subspecies of Spotted Owls (*Strix occidentalis*) where they come into close contact in northern California, USA. Spotted Owls occur in forested habitat in western North America from British Columbia south to the Baja Peninsula and Michoacán in central Mexico (Gutiérrez, Franklin, & Lahaye, 1995). Three subspecies exist, each receiving varying levels of legal protection. Mexican Spotted Owls (*S. o. lucida*) possess the largest range and reside in southern Utah, Colorado, Arizona, New Mexico, Texas, and parts of northern and central Mexico. Mexican Spotted Owls are protected as threatened under the U.S. Endangered Species Act (U.S. Fish and Wildlife Service 1993). Northern Spotted Owls (NSO; *S. o. caurina*), also federally listed as threatened (U.S. Fish and Wildlife Service 1990), inhabit old growth forests from southwestern British Columbia, western Washington, western Oregon, and northwestern California. California Spotted Owls (CSO; *S. o. occidentalis*) occur in the southern Cascade range in northern California, the Sierra Nevada Mountains, and mountainous regions in central and southern California (Davis & Gould, 2008). They are currently listed as a Bird Species of Special Concern in California (Davis & Gould, 2008), and petitions are currently being evaluated to determine whether the subspecies warrants protection under the U.S. Endangered Species Act (U.S. Fish and Wildlife 2015). Numerous morphological and plumage characters have been suggested for distinguishing Spotted Owl subspecies (Barrows, Bloom, & Collins, 1982; Pyle, 1997; Ridgeway, 1914); however, more recent analyses have failed to unambiguously diagnose CSO and NSO (Courtney et al., 2004).

Although morphological characters do not clearly distinguish Spotted Owl subspecies in all cases, genetic analyses indicate that Spotted Owl subspecies are well‐differentiated from one another (Barrowclough & Gutiérrez, 1990; Barrowclough, Gutiérrez, & Groth, 1999; Haig, Mullins, & Forsman, 2004). However, despite clear differentiation, evidence exists for introgression and hybridization among subspecies. These inferences come from analyses that lacked samples from landscape regions where the subspecies come into closest contact (Barrowclough, Groth, Mertz, & Gutiérrez, 2005; Funk, Forsman, Mullins, & Haig, 2008; Haig, Mullins, & Forsman, 2004) or are based solely on mitochondrial DNA sequences (mtDNA) (Barrowclough, Gutiérrez, Groth, Lai, & Rock, 2011), thereby limiting their ability to identify and quantify hybridization patterns in detail. In this investigation, we used new NSO and CSO samples from northern California where the two subspecies come into close proximity, thereby allowing us to make more refined inferences about interactions between Northern Spotted Owls and California Spotted Owls. By placing data from our new samples (10 microsatellite loci and mitochondrial DNA sequence data) in the context of results from previous range‐wide studies of Spotted Owls (Funk et al., 2008; Haig, Mullins, & Forsman, 2004), our new analyses allow us to (1) make refined inferences about genetic diversity and differentiation patterns of Spotted Owls, particularly in northern California, and (2) characterize hybridization and introgression patterns where NSO and CSO come into close contact.

## 2 MATERIALS AND METHODS

### 2.1 Sample collection

**Figure 1:**
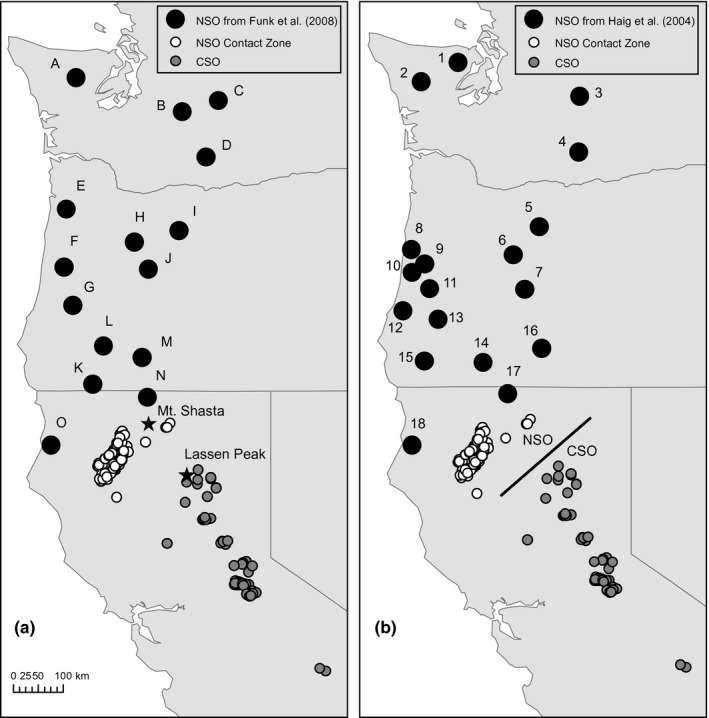
Maps highlighting sampling locations of northern Spotted Owls (NSO) and California Spotted Owls (CSO) used in this study. Panel a reflects samples used for analyses based on microsatellite markers, with locations A through O indicating previously analyzed data reported in Funk et al. (2008). Panel b reflects samples used for analyses of mitochondrial DNA sequences with locations 1 through 18 reflecting data and sample groupings from Haig, Mullins, & Forsman (2004). Small gray circles represent individual California Spotted Owl samples. Small open circles indicate new northern Spotted Owl samples from the northern California contact zone. Pertinent mountain peaks in northern California are highlighted in panel a, whereas the approximate boundary between the ranges of northern Spotted Owls and California Spotted Owls is portrayed in panel b (as per Barrowclough et al., 2011; Gutiérrez & Barrowclough, 2005)

Feather samples from Northern Spotted Owls (*n *=* *126) and California Spotted Owls (*n *=* *105) were collected between 2011 and 2015 from Sierra Pacific Industries forest properties in northern California (Figure 1). At least two feathers were obtained for each sample. All sampling was performed under U.S. Geological Survey banding permit #22568, U.S. Fish and Wildlife Service recovery permit #TE80705A‐1, and California Fish and Wildlife scientific collecting permit #011963. Feathers were individually stored in envelopes and sent to the USGS Conservation Genetics Lab in Corvallis, OR for processing and genetic analyses.

### 2.2 DNA extraction, microsatellite genotyping, and mitochondrial DNA sequencing

DNA extractions were performed in a dedicated clean laboratory in an AirClean Workstation which was decontaminated using UV irradiation and a 10% bleach solution. Aerosol‐resistant pipette tips were used throughout the process and all scissors, blades, and forceps were sterilized in a 50% bleach solution and then rinsed in sterile ddH_2_O between sample preparations. The entire calamus tip of each feather was removed and decontaminated by soaking for 30 min in 70% ethanol, a rinse in ddH_2_O, and then a final 30‐min soak in ddH_2_O. Extractions were performed using QIAGEN DNeasy Blood and Tissue Kit, with incorporated modifications for feather extraction. Two 2.5‐cm calamus shafts were incubated for each bird, with the proteinase K/DTT incubation time increased to 48 hrs with an addition of 20 μl of 20 mg/ml proteinase K added at the 24‐hr time point. Samples were eluted in 100 μl of buffer AE heated to 70°C after a 5‐min incubation. All PCRs were run with appropriate positive and negative amplifications to control for any systematic laboratory contamination. Taxon‐specific primers were used to help exclude contamination sources from common exogenous animal species.

Microsatellite PCR and fragment analyses were performed as previously described in Funk et al. (2008). Owls were genotyped at 10 variable microsatellite loci developed for Mexican Spotted Owls (loci: 6H8, 15A6, 13D8, and 4E10.2; Thode, Maltbie, Hansen, Green, & Longmire, 2002), Lanyu Scops Owls (*Otus elegans botelensis*; loci: Oe3‐7, Oe53, Oe128, Oe129, and Oe149; Hsu, Severinghaus, Lin, & Li, 2003; Hsu, Li, Lin, & Severinghaus, 2006), and Ferruginous Pygmy‐Owls (*Glaucidium brasilianum*; locus: FEPO5; Proudfoot, Honeycutt, & Slack, 2005). PCR conditions and annealing temperatures were the same as those described in the original publications. Locus Oe128 is also a diagnostic marker capable of distinguishing between Spotted Owls and Barred Owls (*Strix varia*; Funk, Mullins, Forsman, & Haig, 2007). Genotypes from all individuals were checked to ensure that no Barred Owls were accidentally included in analyses or that no NSO/Barred Owl hybrids were present.

DNA from a 524‐bp portion of the mitochondrial control region was amplified and sequenced as previously described (Haig, Mullins, & Forsman, 2004). Briefly, primers N1 and D16 (Barrowclough et al., 1999) were used to generate a 1.1‐kb fragment, and internal primers D11 (Barrowclough et al., 1999) and BO24 (Haig, Mullins, & Forsman, 2004) were then used to generate sequence for domain I through a portion of domain II in the control region. All PCR products were bidirectionally sequenced with BigDye version 3.1 dye terminator sequencing chemistry and resolved on an ABI 3730 automated DNA sequencer. Resulting sequence chromatograms were aligned, edited, and trimmed using the programs Geneious 7.0.6 (Kearse et al., 2012) and BioEdit 5.0.1 (Hall, 1999). The program FaBox (Villesen, 2007) was used to facilitate identification of unique mtDNA haplotypes and to generate input files for many of the mtDNA‐based analyses described below.

### 2.3 Integrating data with data from previous studies

New data from the 10 microsatellite loci were integrated with existing data from 352 NSO samples (locations A through O of Funk et al., 2008) and 23 CSO samples (locations P and Q from Funk et al., 2008), resulting in a final data set containing 471 NSO and 127 CSO individuals. All CSO samples were treated as a single location in our new analyses given the mostly continuous distribution of individual sampling sites (Figure 1). mtDNA data from new samples were combined with existing data for 131 NSO individuals (locations 1 through 18 from Haig, Mullins, & Forsman, 2004; which also included previous data from Barrowclough et al., 1999) and 27 CSO individuals (locations 19 and 20 of Haig, Mullins, & Forsman, 2004) to create a final data set with 250 NSO and 130 CSO individuals. As with the microsatellite data, we treated all CSO samples as a single location in analyses when appropriate (Figure 1). Samples for prior investigations (Funk et al., 2008; Haig, Mullins, Forsman, Trail, & Wennerberg, 2004) were collected between 1990 and 2006. With an average generation time of ~10 years in Spotted Owls (Noon & Biles, 1990), the variation in collection dates suggests that there would be at most a ~3‐generation gap between the oldest and most recent samples, which would provide little opportunities for genetic drift to influence genetic structure in this system.

Our new sample set included a large number of individuals from the southernmost extent of the NSO range in northern California (Figure 1). Because these samples represent the closest locations in our study to the northernmost part of the range of CSO (Gutiérrez & Barrowclough, 2005), we refer to this collection of individuals as the “NSO contact zone” in the remainder of this manuscript. We distinguish this from the actual contact zone between subspecies, which occurs just north of the northernmost CSO samples included in our study (Figure 1b; see Barrowclough et al., 2011; Gutiérrez and Barrowclough et al., 2011). Our CSO samples are comprised of birds that were sampled across the northern Sierra Nevada range and southern Cascades, which approaches the northernmost extent of the subspecies' distribution. Although a sampling gap exists in our study between the closest samples from each subspecies, note that juvenile NSO have been reported to disperse distances >100 km (Forsman et al., 2002; Gutiérrez, Franklin, Lahaye, Meretsky, & Ward, 1985). Detailed range‐wide dispersal distance data for CSO have not been obtained; however, investigations of an insular CSO population in southern California identified juvenile dispersal distances in excess of 35 km (Lahaye et al. 2001). Results of that study likely underestimate range‐wide CSO dispersal patterns as it focused solely on individual movements within a specific small area (Lahaye et al. 2001). Based on these studies, the spatial sampling gap between the ranges of CSO and NSO appears to be small enough to be traversed by a Spotted Owl in a single generation.

### 2.4 Genetic diversity and differentiation patterns

We used Arlequin version 3.5 (Excoffier & Lischer, 2010) to quantify genetic diversity among sampling locations for the purposes of comparing results for the new sample sets (NSO contact zone and CSO) to results obtained from prior analyses (locations 1–18 in Haig, Mullins, & Forsman, 2004; locations A through O of Funk et al., 2008). For the mtDNA data, we calculated haplotype diversity (*H*), nucleotide diversity (π), and number of unique haplotypes detected (*A*) for each location. For the microsatellite data, we calculated expected heterozygosity (*H*_*e*_) and the average number of alleles per locus (*A*).

We used the AMOVA procedure (Excoffier, Smouse, & Quattro, 1992) to quantify genetic differentiation patterns among all pairwise combinations of sampling locations for each data set. For the microsatellite data, all sampling locations illustrated in Figure 1a were used, treating the sets of CSO samples and NSO contact zone samples as sampling locations in addition to locations A through O from Funk et al. (2008). For the mtDNA data, analyses were performed using the number of nucleotide differences between haplotypes as the distance measure and included all sampling locations illustrated in Figure 1b, again treating the CSO and NSO contact zone samples as separate sampling locations. In all analyses, the significance of pairwise Φ_ST_ (mtDNA) or F_ST_ (microsatellites) values were obtained using 5,000 randomization replicates. Interpretation of the pairwise Φ_ST_ and *F*_ST_ matrices was difficult due to the large numbers of pairwise comparisons involved in each data set. We therefore used MEGA version 7.020 (Kumar, Stecher, & Tamura, 2016) to generate neighbor‐joining (NJ) trees (Saitou & Nei, 1987) and visualize the pairwise differentiation matrices derived for each data set. In NJ analyses, negative values of Φ_ST_ or F_ST_ between locations were treated as zeros.

Genetic structure patterns from the microsatellite data were also identified using STRUCTURE version 2.3.4 (Pritchard, Stephens, & Donnelly, 2000). Analyses were performed using all genotyped NSO and CSO individuals and were based on ten replicates each of assumed values of *K* (number of genetic clusters) ranging from 1 to 5. Analysis parameters included use of the admixture and correlated allele frequency models, as recommended by Falush, Stephens, and Pritchard (2003), along with 2 × 10^6^ burnin steps and 10^7^ Markov Chain Monte Carlo steps. We used the Δ*K* method of Evanno, Regnaut, and Goudet (2005) to infer the most likely number of genetic clusters associated with the data and likewise used the program CLUMPP (2007) to combine results across replicates for the inferred *K* value.

Phylogenetic structure among unique mtDNA sequence haplotypes was investigated using two approaches. Bayesian phylogenetic analyses (Yang & Rannala, 1997) were based on the program MRBAYES version 3.2.6 (Ronquist et al., 2012). Analyses were performed using the HKY+I nucleotide substitution model as identified using jModelTest2 (Darriba, Taboada, Doallo, & Posada, 2012). MRBAYES analyses were implemented using four runs with the following parameters: 5,000 sampled trees recorded every 2,000 generations with the initial 1,000 trees discarded as burnin. The standard deviation of splits frequencies was used to determine whether convergence among runs was occurring. Maximum‐likelihood analyses were implemented using RAxML version 8.2.4 (Stamatakis, 2014) assuming an invariant sites model, with node support for the best tree assessed using 1,000 bootstrapping replicates. Resulting trees were visualized using MEGA 7.020 (Kumar et al., 2016).

### 2.5 Hybridization and gene flow between subspecies in northern California

Patterns of hybridization and introgression were investigated for the microsatellite data using the Bayesian approach of Anderson and Thompson (2002) as implemented in the program NEWHYBRIDS (https://github.com/eriqande/newhybrids; accessed 21 December 2016). We used this analysis to obtain the posterior probability that each individual fell into one of six different categories: pure CSO, pure NSO, F1 hybrid, F2 hybrid, CSO backcross, and NSO backcross. Five replicate analyses were performed with unique random number seeds, with each replicate implemented using 5 × 10^5^ Markov chain Monte Carlo steps recorded after an initial 5 × 10^4^ burnin steps and Jeffrey's priors on π and θ. After analyses were completed, we identified the category with the highest average posterior probability for each individual and considered an individual's hybrid status as “unknown” if the highest posterior probability was <0.5.

In the case of NSO samples, we quantified the spatial distribution of detected hybrids and pure individuals by generating a 5 × 2 contingency table (five detected NSO classifications × two locations: the NSO contact zone vs. the remainder of the NSO range). The primary goal of this analysis was to determine whether hybrid individuals were overrepresented in the NSO contact zone relative to other NSO regions. The contingency table was analyzed using the “fisher.test” function in R version 3.3.2 (R Development Core Team 2016) to determine whether difference in the frequencies of hybrid and nonhybrid individuals existed between geographic areas. CSO samples were also analyzed to determine whether spatial variation in the locations of hybrid versus nonhybrid individuals existed. In this case, we evaluated the hypothesis that nonpure CSO individuals (i.e., CSO individuals classified as F2, NSO, or unknown) were identified at more northern locations of the CSO range closer to the NSO contact zone. This analysis was implemented via logistic regression with the “glm” function in R using sample latitude as the independent variable and individual sample classification (pure CSO vs. different nonpure CSO categories) as the response variable.

Our data set included CSO samples that were collected in either 1996 (*n *=* *23; samples from Funk et al., 2008) or 2012–2015 (*n *=* *104), thereby permitting us to evaluate the hypothesis that there has been no change in the frequencies of different hybridization categories during this period. The analysis was performed by constructing a 2 × 3 contingency table that summarized the number of putative CSO samples determined to be either pure CSO, pure NSO, or of hybrid/unknown ancestry for each year. We tested for independence of categories across years using the “fisher.test” function in R. To visualize these results, and also to identify possible differences in locations of hybrids and nonhybrids that may occur between older (1996) and newer (2012–2015) CSO samples, we generated plots that displayed the latitudinal position of pure CSO versus non‐CSO samples from each time period.

The relative support for five different gene flow models were quantified for mtDNA and microsatellite data sets using the Bayesian inference framework implemented in program MIGRATE‐N version 3.6.6 (Beerli, 2006). Given our interest in understanding gene flow between NSO and CSO, we restricted this analysis to our NSO samples from the contact zone and the complete set of CSO samples in order to minimize the total number of estimated parameters and maintain focus on the most geographically proximate sets of samples from the two subspecies (Figure 1). The five gene flow models included (1) the full migration model (asymmetric migration between populations), (2) migration only from NSO to CSO, (3) migration only from CSO to NSO, (4) symmetric (equal) migration between subspecies, and (5) no gene flow. For the microsatellite data, analyses under each gene flow model were performed assuming the Brownian motion mutational model, uniform priors on θ ranging from 0 to 30 (δ = 3; 1,500 bins), and uniform priors on *M* ranging from 0 to 100 (δ = 10; 1,500 bins). The Markov Chain Monte Carlo search strategy for each analysis involved recording 2,000 states sampled every 100 steps after an initial burnin of 2 × 10^5^ steps. Twenty concurrent replicates were performed, each based on a static heating scheme with a swapping interval of 1 and 10 chains with temperatures of 1, 1.12, 1.28, 1.49, 1.79, 2.22, 2.94, 4.35, 8.33, and 10^6^. For the mtDNA data, we used the DNA sequence model with a transition/transversion ratio of 6.12 as estimated using the program MEGA (Kumar et al., 2016), uniform priors on θ ranging from 0 to 0.1 (δ = 0.01; 1,500 bins), and uniform priors on *M* ranging from 0 to 10,000 (δ = 1,000; 1.500 bins). Search strategies for analyses were based on 2,000 recorded steps sampled every 100 iterations and 10 concurrent chains, with each chain implementing static heating as described for the microsatellite data. After analyses, we used the reported marginal likelihoods for each model (log‐probability for the mtDNA, Bezier approximations for microsatellites) to compute Bayes factors as per Beerli and Palczewski (2010) and determine the relative support of each model relative to the model with the highest likelihood.

## 3 RESULTS

### 3.1 Genetic diversity and differentiation

Genetic diversity varied among populations (Table 1). For the microsatellite data, values of *H*_*e*_ ranged from 0.685 (location E) to 0.767 (NSO Contact Zone), whereas the average number of alleles per locus (*A*) showed greater variation, ranging from 4.7 (locations D and E) to 7.9 (NSO Contact Zone). Our analyses of mtDNA sequences identified 101 unique haplotypes among the 380 individuals examined (GenBank accession numbers MF187108–MF187208). Diversity revealed by the mtDNA data was relatively high, with haplotype diversity (*H*) ranging from 0.7 (locations 2, 8, 10, and 11) to 1.0 (location 5) and nucleotide diversity ranging from 0.0008 (location 7) to 0.0192 (location 17).

**Table 1:** Sample sizes and genetic diversity parameters for analyses of microsatellite data and mitochondrial DNA sequences (mtDNA) in samples of Northern Spotted Owls and California Spotted Owls. Locations A through O in the microsatellite analyses refer to locations as analyzed in Funk et al. (2008; Figure 1a). Locations 1 through 18 in the mtDNA analyses refer to locations as analyzed in Haig, Mullins, & Forsman (2004; Figure 1b). Parameters listed include sample sizes (*n*), expected heterozygosity (*H*_*e*_), average number of alleles per locus or observed number of haplotypes (*A*), haplotype diversity (*H*), and nucleotide diversity (π)

Microsatellites	*n*	He	*A*	mtDNA	*n*	*A*	*H*	π
Sampling location				Sampling location				
A‐Olympic	22	0.723	5.4	1‐Quilcene	5	3	0.800	0.0082
B‐WA Western Cascades	13	0.756	5.1	2‐Quinault	5	3	0.700	0.0074
C‐Cle Elum	51	0.747	6.4	3‐Wenatchee	5	4	0.900	0.0074
D‐Yakima	18	0.702	4.7	4‐Yakima	5	4	0.900	0.0086
E‐Northern Coast	12	0.685	4.7	5‐Warm Springs	5	5	1.000	0.0144
F‐Mid‐Coast	47	0.720	6.3	6‐Eugene‐Cascades	10	5	0.756	0.0052
G‐South Coast	31	0.750	5.9	7‐Willamette NF	5	2	0.400	0.0008
H‐NW OR Cascades	15	0.715	5.5	8‐Waldport	5	3	0.700	0.0117
I‐Warm Springs	14	0.727	5.4	9‐Alsea	5	4	0.900	0.0078
J‐West OR Cascades	28	0.748	6.2	10‐Mapleton	5	3	0.700	0.0019
K‐Siskiyous	17	0.760	6.2	11‐Eugene‐Coast	5	3	0.700	0.0121
L‐South Umpqua	10	0.764	5.3	12‐Coos Bay	7	5	0.905	0.0180
M‐South Cascades	32	0.763	6.4	13‐Roseburg	10	7	0.911	0.0140
N‐Klamath	14	0.761	5.6	14‐Jackson Co.	10	8	0.956	0.0193
O‐North CA Coast	28	0.753	6.2	15‐Josephine Co.	8	7	0.964	0.0149
NSO Contact Zone	119	0.767	7.9	16‐Klamath Co.	6	3	0.733	0.0091
CSO	127	0.724	7.7	17‐Klamath NF	10	7	0.911	0.0192
				18‐Humboldt	20	9	0.821	0.0103
				NSO contact Zone	119	39	0.923	0.0144
				CSO	130	31	0.802	0.0062

**Figure 2:**
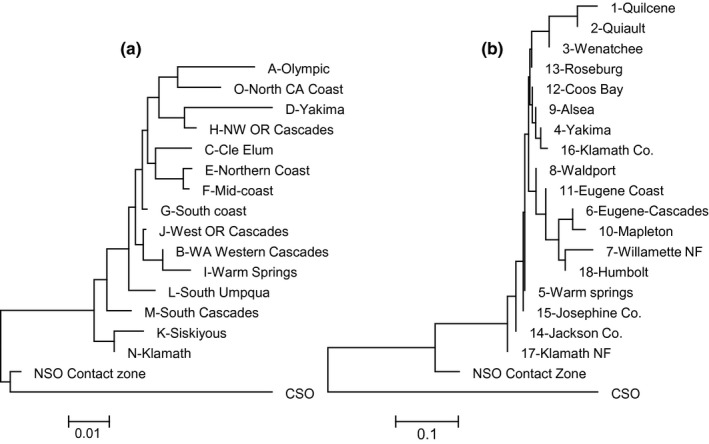
Neighbor‐joining trees summarizing pairwise differentiation patterns among sampling locations for the microsatellite data (panel a) and mtDNA data (panel b). Sample locations correspond to those outlined in Figure 1. Distance matrices used to construct the trees are provided in Tables S1 and S2

**Figure 3:**
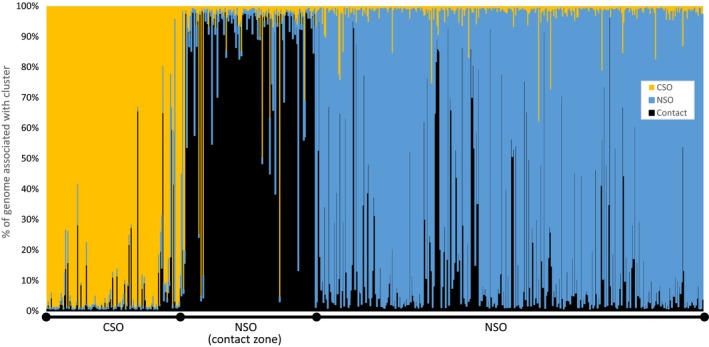
Results of Spotted Owl analyses using STRUCTURE. The program identified *K *=* *3 clusters among the 598 individuals included in analyses. Each analyzed individual is represented by a vertical bar along the *X* axis. The relative amount of shading from each of the three clusters indicates the proportion of each individual's ancestry that was derived from the cluster

Genetic differentiation patterns were consistent between microsatellite and mtDNA data sets. Both indicated highly significant genetic structure among populations (microsatellites: *F*_ST_ = 0.061, *p* < .0001; mtDNA: *F*_ST_ = 0.547, *p *<* *.001). However, substantial variation in differentiation patterns existed in the pairwise comparisons of locations (Tables S1 and S2). As expected, the CSO samples emerged as being most highly divergent from the NSO samples associated with the other locations (Figure 2). Furthermore, NSO in the Contact Zone appeared to be intermediate in divergence between the CSO samples and the other NSO samples from outside the Contact Zone (Figure 2). Results of analyses using STRUCTURE reiterated this pattern (Figure 3). The analysis suggested the presence of *K *=* *3 genetic clusters based on the Δ*K* method, and those clusters corresponded to the sets of CSO samples, NSO Contact Zone samples, and NSO samples from the remainder of the subspecies' range.

Our phylogenetic analyses of the mtDNA sequence data mirrored results of STRUCTURE analyses and provided insights regarding the intermediate differentiation of the NSO Contact Zone samples relative to other NSO locations and the CSO samples. Bayesian (Figure 1) and ML (Fig. S1) analyses produced relatively similar results and identified the same general haplotype groups. The analyses differed primarily in the arrangement of haplotypes within groups of interest and in the level of node support provided by posterior probabilities versus bootstrap values (Bayesian posterior probabilities are expected to be greater than bootstrap proportions: Alfaro, Zoller, & Lutzoni, 2003; Erixon, Svennblad, Britton, & Oxelman, 2003). In Bayesian analyses, the average standard deviation of split frequencies was 0.0076, highlighting the congruence of results across independent runs. In general, the analysis identified a set of haplotypes found primarily (but not exclusively) in CSO along with separate clades containing haplotypes predominantly from NSO. Haplotype sharing between subspecies was noted (haplotypes H3, H8, H87, and H20). Furthermore, both analyses identified a related group of haplotypes found solely among samples from the NSO contact zone.

### 3.2 Hybridization and gene flow

Analyses based on NEWHYBRIDS revealed evidence of introgression (Table 2). Of the 127 putative CSO samples analyzed, 103 (81.1%) were identified as pure CSO, with the remainder categorized as F2s, NSO backcrosses, pure NSO, or unknown. Of the 471 putative NSO samples, 367 (77.9%) were categorized as pure NSO, with the remainder identified as pure CSO, F2s, NSO backcrosses, or of unknown status. No individuals from either subspecies were categorized as an F1 or CSO backcross. Among putative NSO samples, our analysis indicated that hybrid individuals were overrepresented in the NSO contact zone (Table 3). Of the 352 individuals from main NSO range, only eight (2.3%) were categorized as a hybrid. By contrast, of the 119 individuals from the contact zone, 66 individuals (55.5%) were categorized as either an F2 or NSO backcross. *p*‐values from the contingency table analysis were highly significant (*p *< 10^‐5^). The incidence of inferred hybrids among NSO contact zone samples (55.5%) was substantially greater than the frequency of hybrids among CSO samples (18.9%; Table 2).

**Table 2:** Results of analyses of 10 microsatellite loci with the program NEWHYBRIDS. Each of the 598 Spotted Owl samples in our data set was classified into one of six different hybridization categories

Original designation	*n*	Number of individuals assigned to each category						
		CSO	CSO backcross	F1	F2	NSO backcross	NSO	Unknown
CSO	127	103	0	0	18	0	2	4
NSO	471	3	0	0	64	8	367	29

**Table 3:** Geographic location of NSO individuals classified into different hybridization categories using 10 microsatellite loci. “Contact Zone” refers to the set of NSO samples from northern California as indicated in Figure 1. “Main Range” refers to locations A through O as illustrated in Figure 1a

Classification of NSO sample	Contact zone	Main range
NSO	35	332
F2	56	8
NSO backcross	8	0
CSO	3	0
Unknown	17	12
Total	119	352

In contrast to the NSO samples, our logistic regression analysis indicated no evidence that CSO samples classified as CSO hybrids, NSO, or unknown were aggregated in more northern parts of the CSO range (all nonpure CSO categories combined: *z* = 0.715, *p *=* *.474; F2: *z* = −0.429, *p *=* *.668; NSO: *z* = 0.885, *p *=* *.376; Unknown: *z* = 1.293, *p *=* *.196). Our temporal analyses of CSO samples suggested that hybrid or misclassified individuals were more prevalent in 1996 samples than in 2012–2015 samples (Table 4). Among samples from 1996, 48% were inferred to be of hybrid or unknown status, whereas only 10% of samples from 2015 were similarly classified. Likewise, the two NSO individuals misidentified as CSO were observed in 1996, whereas no NSO individuals were detected among the putative CSO samples in 2015. Fisher's exact test deemed these differences to be highly significant (*p *=* *.0005). Although this temporal difference existed, the spatial distributions of pure CSO individuals were similar to the distributions of non‐CSO individuals for each time period that CSO samples were collected (Fig. S2) and reiterated the absence of a spatial pattern revealed by the logistic regression analyses.

**Table 4:** Comparison of inferred hybrid status (CSO: California Spotted Owl; NSO: Northern Spotted Owl; or Hybrid/unknown) between putative CSO samples collected in 1996 versus 2015

	Collection year	
Category	1996	2015
CSO	10	93
Hybrid/Unknown	11	11
NSO	2	0
Total	23	104

Various gene flow models were evaluated using the program MIGRATE. In agreement with results from NEWHYBRIDS that identified a greater incidence of hybrids in the NSO contact zone relative to CSO samples, both microsatellite and mtDNA data ranked the five models the same way and produced overwhelming evidence indicating that the direction of migration was predominantly from CSO into the NSO Contact Zone (Table 5). Models that allowed for asymmetric migration were a distant second, whereas the model allowing for no gene flow received the lowest support.

**Table 5:** Results of analyses using MIGRATE to infer the relative support for five migration models with the mitochondrial DNA sequence data (mtDNA) and 10 microsatellite loci

	Migration model	Marginal likelihood	Bayes factor	Model rank
mtDNA	Asymmetric	−1,971.38	−18.36	2
	NSO ‐> CSO	−1,978.59	−32.77	4
	CSO ‐> NSO	−1,962.20	0.00	1
	Symmetric	−1,976.12	−27.84	3
	None	−2,152.23	−380.06	5
Microsatellites	Asymmetric	−38,180.17	−9,419.31	2
	NSO ‐> CSO	−41,852.98	−13,092.12	4
	CSO ‐> NSO	−28,760.86	0.00	1
	Symmetric	−39,936.60	−11,175.74	3
	None	−328,209.26	−299,448.40	5

## 4 DISCUSSION

### 4.1 Genetic differentiation of Spotted Owls in northern California

Our analyses provide a refined understanding of genetic differentiation and introgression patterns of Spotted Owls in northern California. Among NSO populations, genetic differentiation patterns have been identified and discussed using increasing numbers of samples and with different genetic marker systems over time (Barrowclough & Gutiérrez, 1990; Barrowclough et al., 1999, 2005; Funk et al., 2008; Haig, Wagner, Forsman, & Mullins, 2001; Haig, Mullins, & Forsman, 2004). Some general patterns that emerged include the identification of significant genetic structure characterized by an isolation‐by‐distance pattern in analyses of nuclear genetic markers (RAPDs: Haig et al., 2001; microsatellites: Funk et al., 2008), but not in analyses of mtDNA (Haig, Mullins, & Forsman, 2004). Measures of genetic differentiation reported in these studies were variable, but pointed toward relatively similar overall conclusions (Table 8 in Haig, Mullins, & Forsman, 2004: average pairwise *F*_*ST*_ of NSO was 0.014 for mtDNA; Table 1 in Funk et al., 2008: average pairwise *F*_*ST*_ of NSO = 0.025 for microsatellites). In this study, we added substantial new data to these prior data sets, particularly in the range of the NSO in northern California (Figure 1). When placed in the context of existing data from NSO populations, the NSO contact zone population showed substantially higher differentiation for both mtDNA and microsatellite data (Figure 2). In this case, the average *F*_*ST*_ between the contact zone and other NSO populations ranged from 0.034 to 0.079 for the microsatellite data (Table S1), whereas congruent values for mtDNA ranged from 0.167 to 0.268 (Table S2).

**Figure 4:**
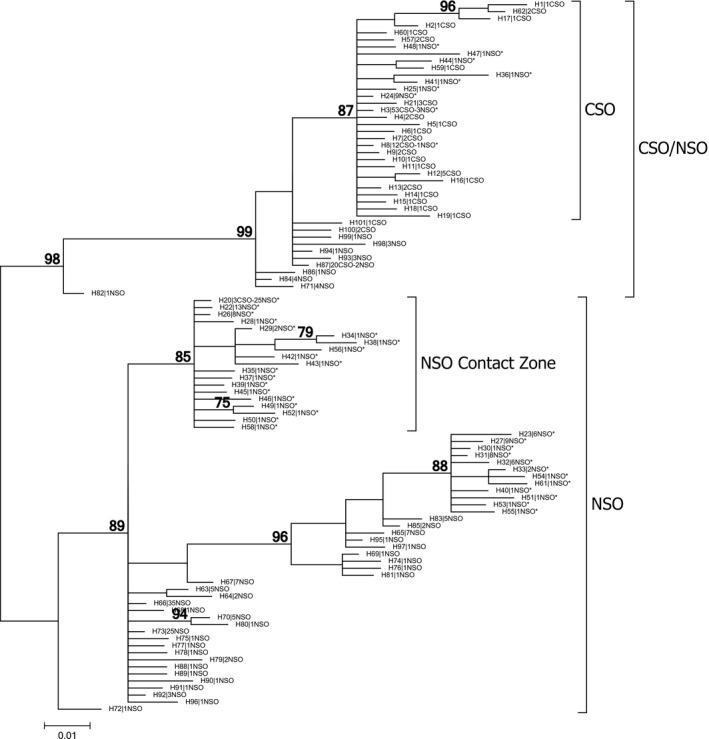
Phylogenetic analyses of 101 unique mtDNA haplotypes using Bayesian inference. Nodes with posterior probabilities ≥0.75 are indicated for the Bayesian analysis. Terminal node labels on the Bayesian tree indicate the number of CSO or NSO samples identified with each haplotype (e.g., H8|12CSO‐1NSO refers to haplotype H8, which was identified in 12 CSO samples and 1 NSO sample). Nodes indicated with an asterisk identify a haplotype that was detected in the NSO Contact Zone samples

High differentiation in the NSO contact zone population was explained in part by our phylogenetic analyses of the mtDNA (Figure 4). Although many haplotypes from the NSO contact zone were identical or closely related to NSO haplotypes detected in other parts of the subspecies' range, we also detected a unique lineage of haplotypes that were found solely among NSO samples from the contact zone (Figure 4). This pattern is unexpected given the overall high dispersal ability of Spotted Owls (Forsman et al. 2002; Gutiérrez et al., 1985; Lahaye et al. 2001) and suggests that this region of northern California has a history of isolation that led to the evolution of this previously undocumented lineage. Barrowclough et al. (2011) obtained substantial mtDNA sequence data from northern California, but likely did not resolve the clade because range‐wide NSO data were not included in their analysis. Our STRUCTURE analyses (Figure 3) reiterated the mtDNA phylogeny by identifying the contact zone population as a third, highly differentiated gene pool that was separated from CSO and the main NSO range. Combined, these results suggest that Spotted Owls in northern California were isolated from other Spotted Owls at some point during the evolutionary history of *S. caurina* in western North America. Northern California has been recognized as the location of the Cascade–Sierran suture zone (Remington, 1968) and is associated with high degrees of genetic diversity and phylogeographic breaks associated with historical vicariance events (Swenson & Howard, 2005). In particular, the California Cascades area of northern California is in many respects a naturally isolated landscape region, with the Shasta Valley and the Sacramento River Valley providing wide divisions with unsuitable habitat between the areas that we investigated and other proximate areas with Northern Spotted Owl habitat in the Oregon and California Klamath provinces (see maps in California Department of Fish and Wildlife 2016). Future analyses that incorporate landscape genetics concepts (Manel, Schwartz, Luikart, & Taberlet, 2003) may be better able to help identify the physical aspects of the Northern California region that led to the high level of differentiation detected in this study.

As with Barrowclough et al. (2005, 2011), our phylogenetic analyses independently identified numerous NSO haplotypes that were more closely allied to haplotypes typically found in CSO (Figure 4), whereas CSO haplotypes were restricted to a single lineage. We concur with prior interpretations of this pattern (Barrowclough et al., 2005), which point to incomplete lineage sorting in NSO due to insufficient time for NSO lineages to coalesce given the effective population size of the taxon.

### 4.2 Hybridization pattern between Northern Spotted Owls and California Spotted Owls

Results of our analyses provide the most definitive insights to date regarding introgression and hybridization in Spotted Owls and particularly highlight the high degree of introgression that is occurring in the northern California contact zone. Barrowclough et al. (1999) analyzed mtDNA data from Spotted Owls and suggested that gene flow among subspecies was minimal based on the strong association between haplotypes and subspecific identifications. The Barrowclough et al. (1999) study included a sample from only 10 NSO individuals from northwestern California, but nonetheless revealed some evidence of gene flow based on detection of a single individual that possessed haplotypes identical to those detected in CSO from the northern Sierra Range. Haig et al. (2001), using mtDNA data from a larger set of samples, also identified clades that were largely associated with NSO, CSO, and Mexican Spotted Owl haplotypes and samples. However, CSO haplotypes were detected in approximately 13% of the NSO samples analyzed by Haig et al. (2001), with over 20% of individuals from the Klamath region possessing a CSO haplotype. This result led (Haig, Mullins, & Forsman, 2004; to suggest that a stable Spotted Owl hybrid zone existed in northern California. Barrowclough et al. (2005) performed new mtDNA‐based analyses with increased sampling and confirmed the inferences made by Haig, Mullins, & Forsman (2004) with respect to the existence of a stable hybrid zone, and further provided evidence for bidirectional gene flow between subspecies. Funk et al. (2008) used 10 microsatellite loci and over 350 individuals to investigate genetic structure in Spotted Owls. The Funk et al. (2008) analysis included samples from only 23 CSO individuals, but nonetheless also identified separate subspecific gene pools based on the Bayesian clustering procedure in STRUCTURE (Pritchard et al., 2000), and likewise provided evidence for gene flow and introgression.

All of the above studies lacked detailed sampling from the northern California contact zone and were therefore limited in their ability to rigorously quantify genetic exchange where the two subspecies come into closest contact. This led Barrowclough et al. (2011) to analyze mtDNA from more samples in northern California and identify an area between the Pit River and Lassen Peak as the transition point between landscape locations that possess primarily NSO haplotypes versus CSO haplotypes. Although mtDNA can provide insights regarding the maternal lineages of an individual, nuclear markers, such as microsatellites, are necessary to explicitly identify introgressed or hybrid individuals in a sample (Anderson & Thompson, 2002; Randi, 2008; Vähä & Primmer, 2006) particularly when parental taxa are difficult to distinguish based on morphology or when parents themselves are unknown. Based on mtDNA alone, it becomes difficult to distinguish between a dispersal event that has led to gene flow (and therefore hybridization) versus one that has not (i.e., dispersal without gene flow). Consequently, our new analyses provide the clearest depiction yet of interactions between subspecies owing to the large number of CSO and NSO contact zone samples included in the study (Figure 1) and our use of nuclear genetic markers. In particular, our analyses show that hybrids dominate samples from the NSO contact zone and are found at much lower rates in other parts of the NSO range (Table 3). Compared to the NSO contact zone, hybridization was less apparent among CSO samples and logistic regression analyses suggested that CSO hybrids are not aggregated at the northern edge of the CSO range. Focusing solely on the interaction of CSO and owls from the NSO contact zone, Bayesian analyses provided overwhelming support for a gene flow model dominated by movement from CSO into the NSO contact zone (Table 5). Interspecific hybrids are well known among owls (Order Strigiformes) and different species of *Strix* including (*S. aluco* x *S. uralensis*), (*S. huhla* x *S. nigrolineata*), (*S. hylophila* x *S. rufipes*), and (*S. nebulosi* x *Bubo bubo*) (McCarthy, 2006). In particular, Spotted Owls also hybridize with Barred Owls (*S. varia*; Funk et al., 2007; Haig, Mullins, Forsman, Trail, & Wennerberg, 2004; Hamer, Forsman, Fuchs, & Walters, 1994; Kelly & Forsman, 2004). Consequently, it is unsurprising to obtain evidence for intraspecific hybridization within Spotted Owl as documented in this study.

### 4.3 Hybrid zone dynamics and the absence of F1 hybrids

The dynamics of a hybrid zone can in part be inferred from molecular genetic data (Buggs, 2007). Based on data available at the time, Haig, Mullins, & Forsman (2004) and Barrowclough et al. (2005, 2011) suggested that the Spotted Owl hybrid zone in northern California was stable, with Barrowclough et al. (2005) also suggesting that the zone was actually a “tension zone” (Key, 1968) maintained by a balance between dispersal and selection against hybrids. Given the high degree of differentiation between NSO and CSO, these studies, along with data from our work, also suggest that the hybrid zone is a product of contact between taxa following a prior long‐term isolation event (i.e., a secondary hybrid zone; Barton & Hewitt, 1985). Barrowclough et al. (2011) further suggested that the hybrid zone was symmetric based on relatively equal frequencies of NSO versus CSO haplotypes within 50 km of their proposed transition zone. However, our work identified an extremely high incidence of hybrids in the NSO contact zone (>50%; Table 3) relative to CSO, with most individuals categorized as an advanced hybrid (F2 or backcross). The high incidence of advanced hybrids may indicate that selection against F1 hybrids is minimal given their requirement to produce F2 or backcrossed individuals. Furthermore, rather than being a stable hybrid zone, the greater incidence of inferred hybrids among NSO contact zone samples versus CSO samples suggests that the hybrid zone may be dynamic and moving (Buggs, 2007), in this case northward from the CSO range into the NSO range. Hybrid zones may move for a number of reasons, including differences in density and variation in dispersal between taxa (Barton & Hewitt, 1985). Variation in aggression behavior has also been suggested to influence hybrid zone movement in birds (Pearson, 2000; Pearson & Rohwer, 2000), and climate change is also expected to influence hybrid zone movement (Taylor, Larson, & Harrison, 2015). Future investigations focusing on these and other factors may ultimately be required to determine the specific basis for the strong asymmetry identified in this study.

Although we obtained substantial evidence for the existence of hybrid Spotted Owls in northern California, we found no evidence for any first‐generation hybrids (F1) in our samples. We suggest three nonexclusive hypotheses for this result. First, our results may point to some degree of misclassification of hybrids, which were in most cases identified as F2s in our analyses. Use of additional loci could help resolve F1 individuals from more advanced hybrid categories (Vähä & Primmer, 2006) and help determine whether F1 individuals were systematically categorized as F2s in our analysis. Second, our results may indicate that F1 hybrids exist, but were not sampled because the primary interactions between subspecies that result in F1 hybrids occur elsewhere in northern California. Our NSO contact Zone samples were highly concentrated in an area to the east of the Trinity Alps. However, Northern Spotted Owls are known to occur in many other areas throughout northern California (Barrowclough et al., 2011). It therefore remains feasible that the active center of the hybrid zone lies elsewhere.

Finally, under the third hypothesis, the absence of F1 individuals may indicate that contemporary hybridization is not occurring or that it is occurring at a reduced rate relative to the past. In this case, hybridization could potentially be eliminated or reduced if intervening habitat between the ranges of NSO and CSO has been recently altered such that dispersal no longer occurs or is reduced relative to historical levels. Consistent with this hypothesis, our temporal analyses suggested that the CSO samples from 1996 included a greater proportion of hybrid individuals than the 2015 samples and that NSO were identified among putative CSO samples only in 1996 (Table 4). Numerous changes to the northern California landscape have occurred over the past century as a consequence of logging (Laudenslayer & Darr, 1990; McKelvey & Johnston, 1992), mineral development (Kristofors, 1973), and forest fires (Miller, Safford, Crimmins, & Thode, 2009). However, any change capable of the large‐scale reduction in population connectivity required to invoke this hypothesis would need to be extremely recent given our detection of F2 and backcross hybrids. In that regard, we suggest that recent forest fires in northern California may be most pertinent (Fig. S3). In particular, the Fountain Fire of 1992 may be especially relevant. This fire burned approximately 64,000 acres of forest in the northern Sierra Range (Zhang, Webster, Powers, & Mills, 2008) and also happens to have been located in important intervening habitat between the subspecies' ranges (Fig. S3). The effects of the fire on the landscape are still apparent (Fig. S4) and may be creating a zone of inhospitable habitat that owls do not readily cross. We note, however, that three CSO were identified among the set of NSO Contact Zone samples collected in 2015 (Table 2). This observation indicates that connectivity between subspecies still exists and that F1 hybridization may now be continuing at a reduced (but undetectable) rate relative to the recent past.

### 4.4 Conservation of hybrids

The U.S. Endangered Species Act makes no specific provisions for the protection of hybrids, which has led to changes over time in the perspectives and approaches that federal agencies take when dealing with populations of hybrid organisms (Haig & Allendorf, 2006). Recognizing the need to more formally identify hybrids and issues associated with the management of threatened and endangered taxa, the U.S. Fish and Wildlife Service and National Marine Fisheries Service proposed an intercross policy (USFWS and NMFS 1996) to help provide formal guidance under the many different types of scenarios that hybrids may occur in the wild (Haig & Allendorf, 2006). This policy was not formally approved. However, in the case of hybridization between NSO and CSO, both parental taxa currently receive protection to varying degrees under state and federal conservation laws. Furthermore, given the high rate of hybridization recorded in birds (McCarthy, 2006), the hybridization detected in northern California appears to be a natural process rather than the result of recent anthropogenic influence. Wayne and Shaffer (2016) suggest that protection of hybrids is warranted in these cases.

## AUTHOR CONTRIBUTIONS

SMH directed the research project; EDF contributed samples; MPM and TDM performed analyses; MPM, TDM, EDF, and SMH wrote the manuscript.

## CONFLICT OF INTEREST

None declared.
