# Supplementary material for: Genetic differentiation and inferred dynamics of a hybrid zone between Northern Spotted Owls (Strix occidentalis caurina) and California Spotted Owls (S. o. occidentalis) in northern California
Source: Ecol Evol. 2017 Jul 27;7(17):6871–83. doi: 10.1002/ece3.3260 (PMC5587499; doi:10.1002/ece3.3260)
Supplement: Supplementary file 1 [file ECE3-7-6871-s001.pdf]

Supplemental Table 1. Pairwise measures of genetic differentiation (F_{ST}) between locations for the microsatellite data set. Differentiation measures are provided below the matrix diagonals and P-values are arranged above the diagonal of each matrix. Site locations are provided in Fig. 1.

FST = 0.061, P < 0.0001	A	B	C	D	E	F	G	H	I	J	K	L	M	N	O	NSO Contact Zone	CSO
A. Olympic	-	0.006	0.000	0.000	0.000	0.000	0.002	0.000	0.000	0.000	0.000	0.000	0.000	0.000	0.000	0.000	0.000
B. WA Western Cascades	0.021	-	0.074	0.006	0.035	0.062	0.451	0.062	0.481	0.109	0.000	0.182	0.005	0.046	0.001	0.000	0.000
C. Cle Elum	0.030	0.009	-	0.000	0.005	0.000	0.000	0.000	0.000	0.000	0.000	0.007	0.000	0.008	0.000	0.000	0.000
D. Yakima	0.039	0.026	0.035	-	0.001	0.000	0.000	0.004	0.000	0.001	0.000	0.002	0.000	0.000	0.000	0.000	0.000
E. North Coast	0.044	0.019	0.021	0.040	-	0.283	0.099	0.059	0.029	0.033	0.003	0.020	0.002	0.019	0.002	0.000	0.000
F. Mid. Coast	0.040	0.011	0.014	0.044	0.003	-	0.043	0.000	0.000	0.000	0.000	0.016	0.000	0.005	0.000	0.000	0.000
G. South Coast	0.019	0.000	0.016	0.045	0.011	0.007	-	0.035	0.057	0.058	0.001	0.128	0.005	0.151	0.001	0.000	0.000
H. NW OR Cascades	0.036	0.012	0.032	0.024	0.014	0.026	0.014	-	0.076	0.011	0.000	0.032	0.000	0.001	0.000	0.000	0.000
I. Warm Springs	0.050	0.000	0.037	0.057	0.022	0.033	0.013	0.012	-	0.237	0.000	0.108	0.002	0.003	0.000	0.000	0.000
J. Western OR Cascades	0.040	0.007	0.017	0.023	0.013	0.015	0.008	0.015	0.004	-	0.000	0.117	0.001	0.065	0.000	0.000	0.000
K. Siskiyous	0.058	0.035	0.036	0.057	0.030	0.041	0.026	0.042	0.045	0.027	-	0.131	0.003	0.352	0.000	0.000	0.000
L. South Umpqua	0.041	0.010	0.021	0.040	0.028	0.020	0.012	0.020	0.016	0.010	0.014	-	0.016	0.470	0.006	0.000	0.000
M. S Cascades	0.042	0.020	0.025	0.061	0.027	0.024	0.013	0.035	0.024	0.015	0.022	0.021	-	0.190	0.000	0.000	0.000
N. Klamath	0.050	0.015	0.017	0.050	0.026	0.019	0.007	0.034	0.031	0.009	0.003	0.000	0.006	-	0.002	0.000	0.000
O. Northern CA Coast	0.029	0.029	0.030	0.047	0.026	0.030	0.020	0.029	0.041	0.028	0.028	0.025	0.018	0.024	-	0.000	0.000
NSO Contact Zone	0.069	0.034	0.043	0.079	0.063	0.059	0.042	0.051	0.051	0.044	0.038	0.045	0.036	0.033	0.048	-	0.000
CSO	0.138	0.089	0.107	0.117	0.129	0.118	0.111	0.114	0.121	0.103	0.089	0.118	0.104	0.101	0.106	0.067	-

Supplemental Table 2. Pairwise measures of genetic differentiation (F_{ST}) between locations for the mitochondrial DNA sequence data set. Differentiation measures are provided below the matrix diagonals and P-values are arranged above the diagonal of each matrix. Site locations are provided in Fig. 1.

FST = 0.547, P < 0.0001	1	2	3	4	5	6	7	8	9	10	11	12	13	14	15	16	17	18	19	20
1. Quilcene	-	0.602	0.677	0.757	0.550	0.440	0.414	0.451	0.307	0.251	0.022	0.219	0.317	0.738	0.763	0.719	0.909	0.247	0.790	0.000
2. Quinault	-0.176	-	0.081	0.622	0.315	0.658	0.556	0.455	0.444	0.108	0.014	0.195	0.291	0.657	0.627	0.735	0.582	0.158	0.755	0.000
3. Wenatchee	0.020	-0.067	-	0.519	0.583	0.124	0.205	0.147	0.042	0.559	0.000	0.089	0.282	0.345	0.246	0.202	0.884	0.286	0.284	0.000
4. Yakima	0.140	0.068	-0.126	-	1.000	0.781	0.978	0.297	0.315	1.000	0.029	0.244	0.517	0.779	0.710	0.740	1.000	0.528	0.510	0.000
5. Warm Springs	0.121	0.067	-0.053	-0.054	-	0.224	0.479	0.113	0.059	0.588	0.000	0.079	0.257	0.462	0.836	0.540	0.777	0.287	0.340	0.000
6. Eugene Cascades	0.144	0.034	-0.107	0.024	0.060	-	0.796	0.274	0.788	0.073	0.002	0.111	0.129	0.701	0.662	0.979	0.517	0.068	0.519	0.000
7. Willamette NF	0.299	0.216	0.063	0.178	0.122	0.050	-	0.254	0.367	0.256	0.003	0.145	0.218	0.590	0.764	0.919	0.450	0.229	0.327	0.000
8. Waldport	0.127	0.065	-0.075	-0.024	-0.171	0.023	-0.026	-	0.288	0.172	0.006	0.185	0.196	0.568	0.411	0.394	0.503	0.303	0.618	0.000
9. Alsea	0.109	0.025	-0.175	-0.094	-0.033	-0.063	0.098	-0.050	-	0.053	0.003	0.061	0.101	0.523	0.317	0.630	0.375	0.047	0.489	0.000
10. Mapleton	0.198	0.091	0.048	0.196	0.110	-0.020	0.079	0.017	0.081	-	0.004	0.051	0.276	0.235	0.519	0.182	0.520	0.519	0.119	0.000
11. Eugene Coast	0.071	0.000	-0.078	0.004	-0.172	-0.023	0.018	-0.210	-0.054	-0.034	-	0.002	0.013	0.004	0.024	0.031	0.026	0.003	0.024	0.000
12. Coos Bay	0.084	0.035	-0.043	-0.080	-0.088	0.084	0.089	-0.078	-0.044	0.099	-0.067	-	0.678	0.193	0.095	0.109	0.380	0.053	0.178	0.000
13. Roseburg	0.079	0.019	-0.085	-0.052	-0.105	0.017	0.078	-0.073	-0.064	0.065	-0.087	-0.049	-	0.321	0.188	0.151	0.553	0.087	0.338	0.000
14. Jackson Co	0.116	0.075	0.006	-0.002	-0.117	0.111	0.130	-0.076	0.019	0.120	-0.087	-0.062	-0.052	-	0.731	0.875	0.875	0.188	0.640	0.000
15. Josephine Co	0.122	0.070	-0.013	0.029	-0.141	0.069	0.079	-0.144	0.005	0.064	-0.160	-0.044	-0.041	-0.075	-	0.747	1.000	1.000	0.698	0.000
16. Klamath Co	0.156	0.089	-0.062	-0.080	0.025	0.083	0.158	0.029	-0.034	0.187	0.058	-0.015	-0.048	0.044	0.082	-	0.815	0.172	0.863	0.000
17. Klamath NF	0.148	0.111	0.038	0.020	-0.067	0.157	0.194	0.001	0.050	0.184	-0.009	-0.030	-0.032	-0.075	0.000	0.047	-	0.530	1.000	0.000
18. Humboldt	0.086	0.010	-0.053	-0.001	-0.031	-0.020	-0.004	-0.067	-0.028	-0.034	-0.102	0.019	-0.004	0.036	-0.013	0.058	0.095	-	0.168	0.000
19. NSO Contact Zone	0.268	0.235	0.189	0.191	0.167	0.241	0.271	0.193	0.200	0.264	0.182	0.170	0.169	0.175	0.198	0.197	0.179	0.229	-	0.000
20. CSO	0.818	0.814	0.803	0.801	0.732	0.811	0.830	0.763	0.804	0.825	0.754	0.755	0.750	0.672	0.721	0.812	0.667	0.768	0.643	-

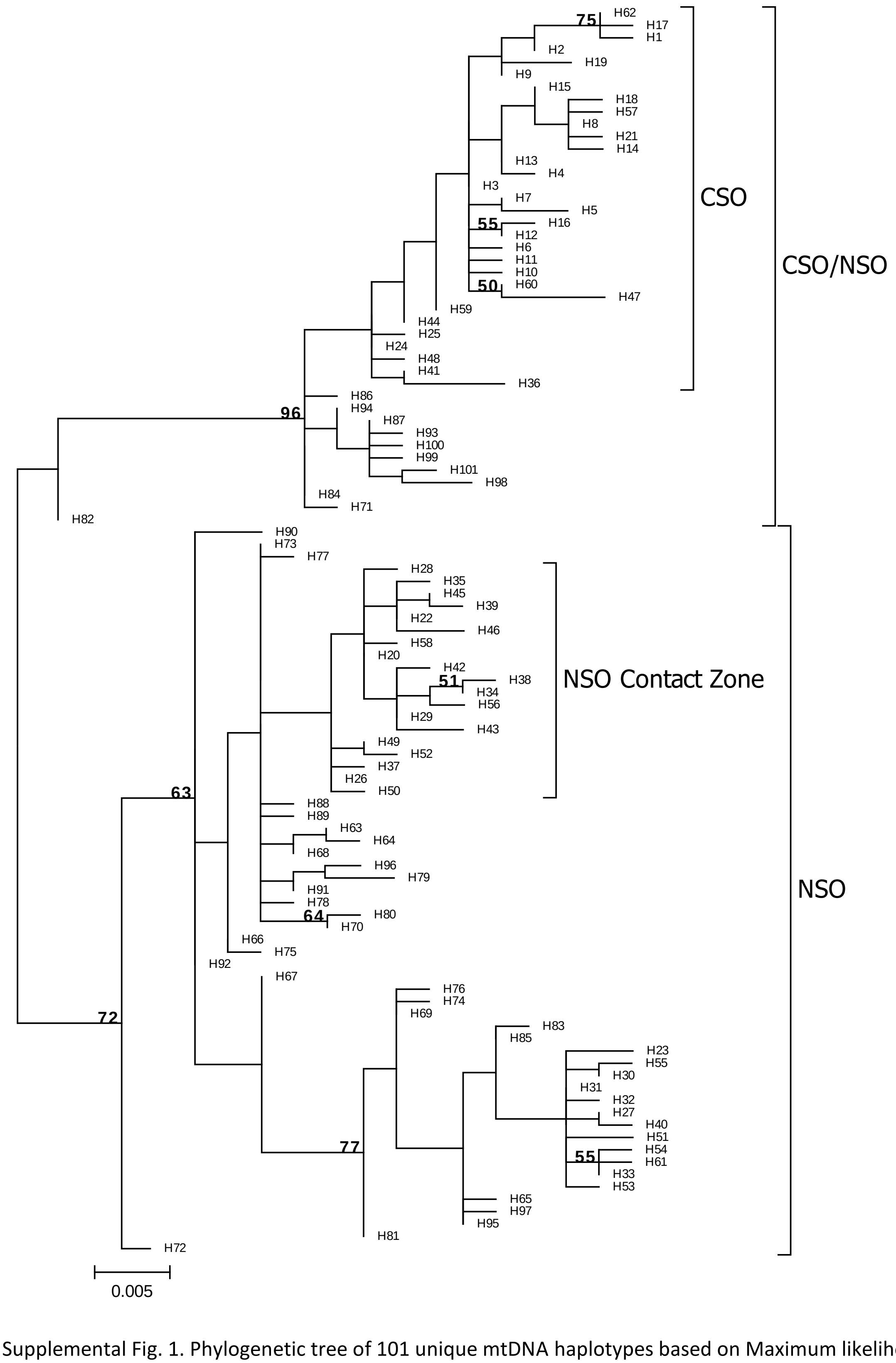

Supplemental Fig. 1. Phylogenetic tree of 101 unique mtDNA haplotypes based on Maximum likelihood inference. Nodes with bootstrap values ≥ 0.5 are indicated. Terminal node labels include haplotype codes presented in Fig. 4.

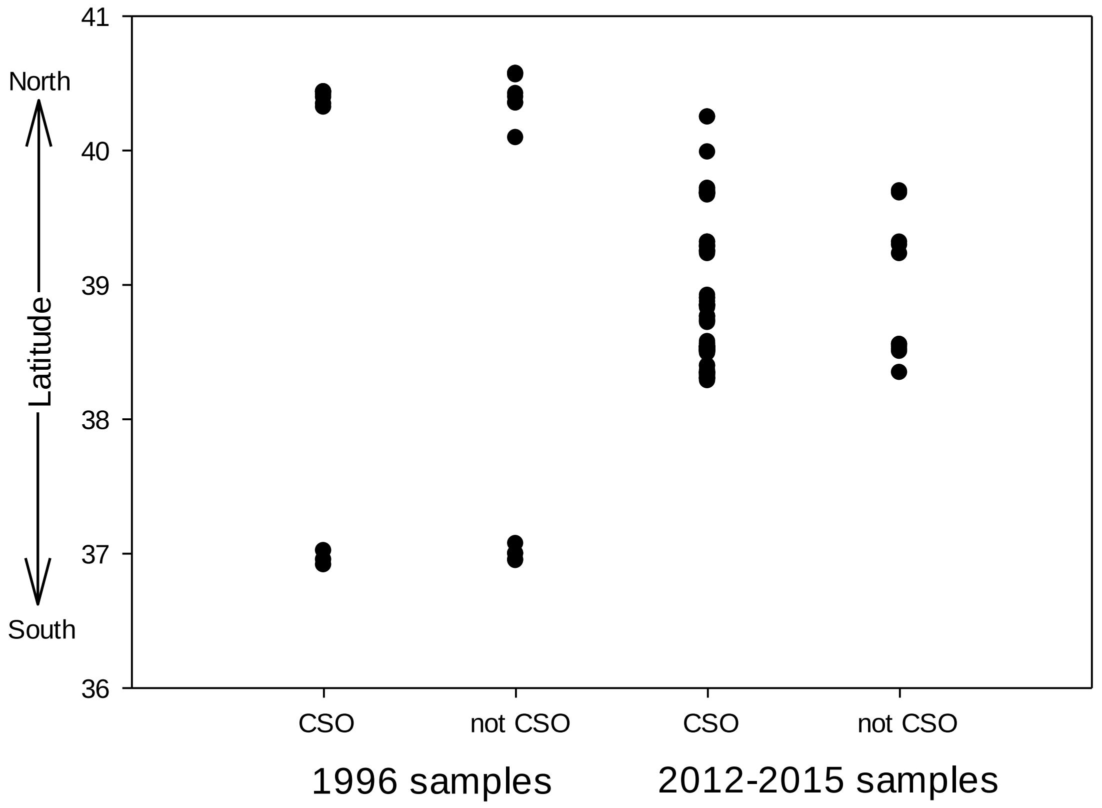

Supplemental Fig. 2. Latitudinal position of samples from the CSO range that were categorized as either pure CSO or not pure CSO for samples obtained in 1996 and 2012-2015.

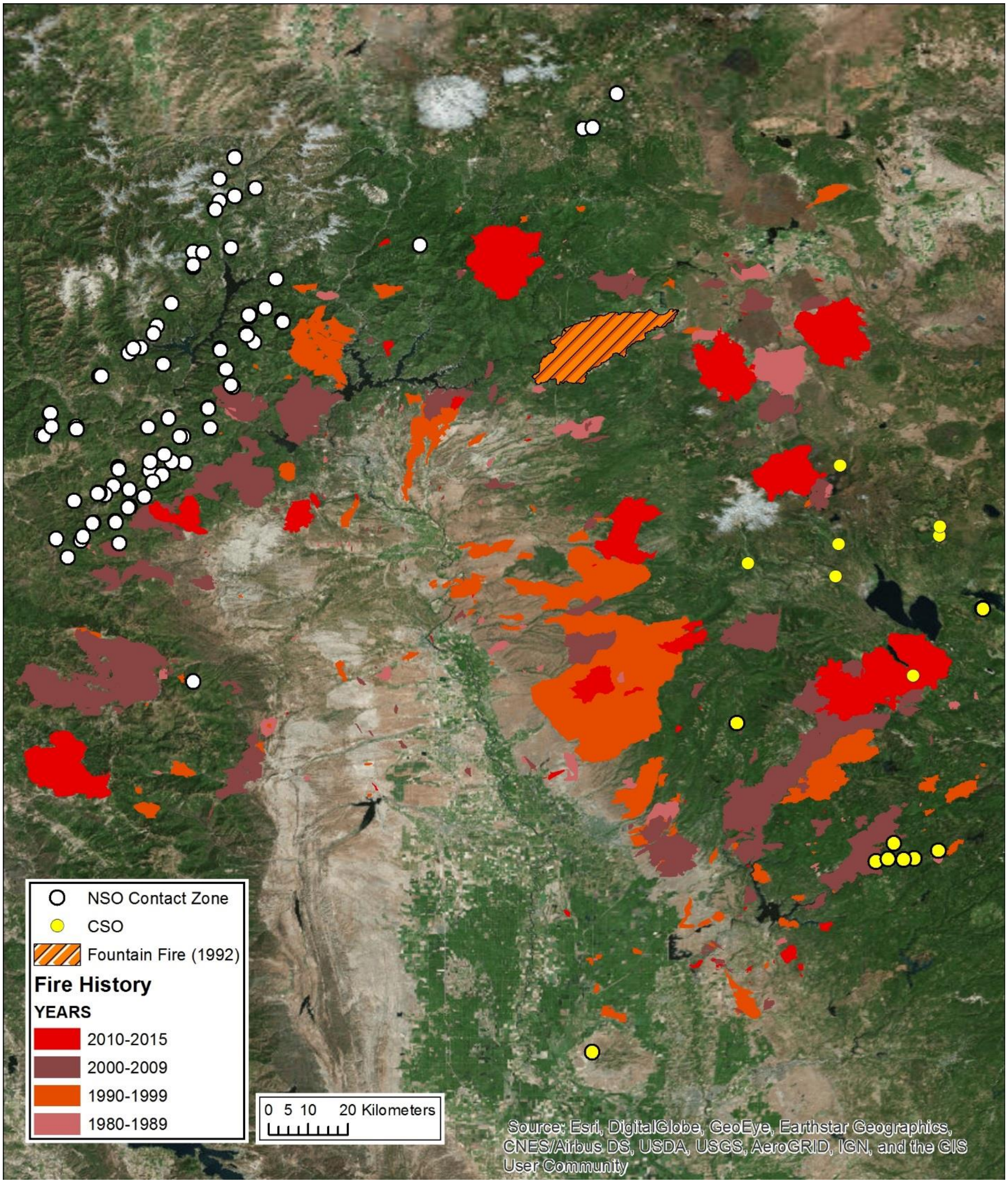

Supplemental Fig. 2. Satellite imagery of northern California from 2014 highlighting the region between NSO Contact Zone samples and CSO samples. Wildfires from the region from 1980 through 2015 are highlighted with shading that reflects the decade of occurrence. The Fountain Fire (1992) is identified separately to facilitate comparisons with Supplemental Fig. 3. Spatial data for fires obtained from http://frap.fire.ca.gov/data/frapgisdata-sw-fireperimeters_download.

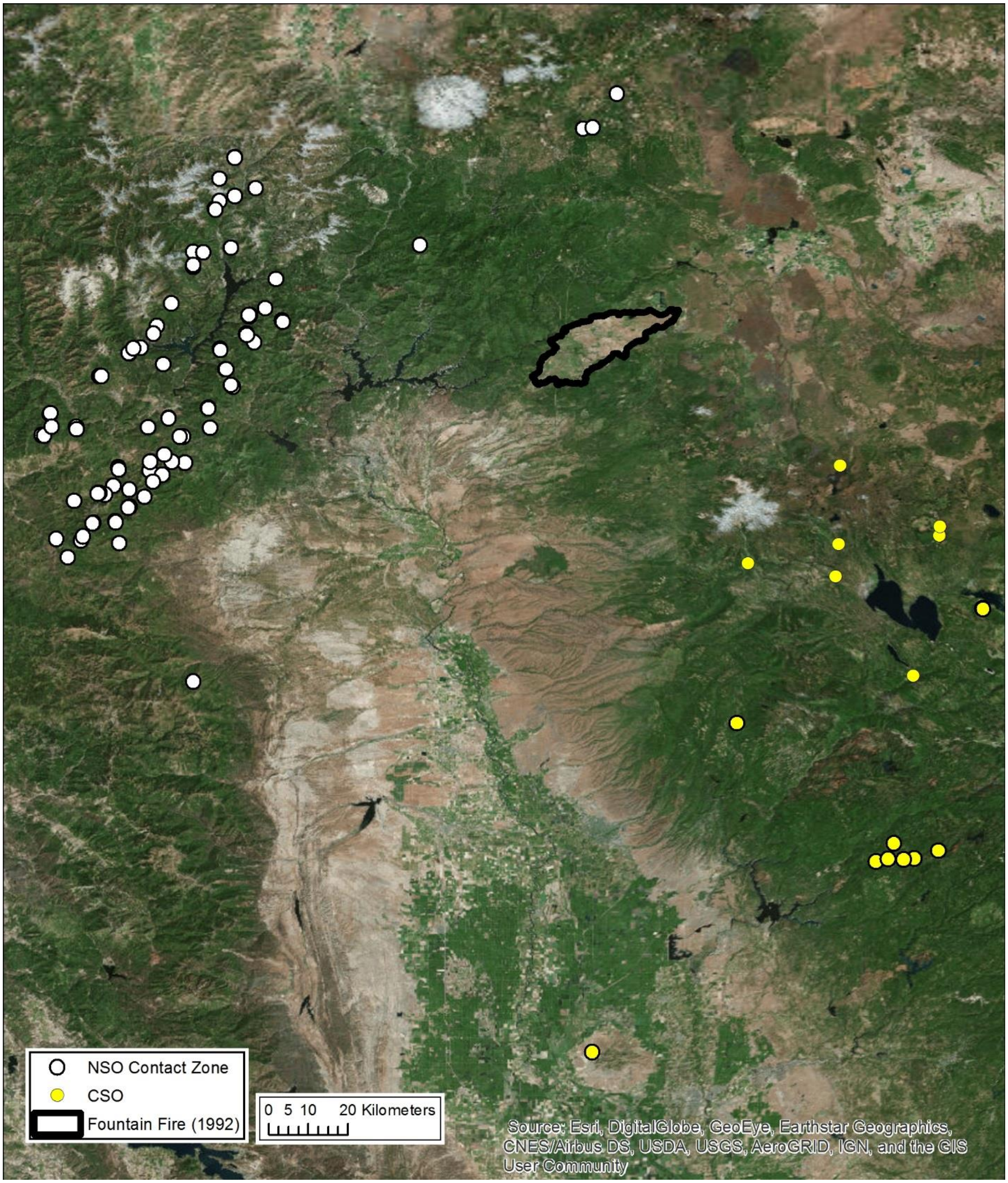

Supplemental Fig 3. Satellite imagery of northern California from 2014 highlighting locations of NSO Contact Zone samples and CSO samples. The boundary of the 1992 Fountain Fire is highlighted to help illustrate the extent of habitat alteration that occurred during the event.
